# Non-antigen-specific Immunoadsorption Is a Risk Factor for Severe Postoperative Infections in ABO-Incompatible Kidney Transplant Recipients

**DOI:** 10.3389/ti.2024.12263

**Published:** 2024-03-14

**Authors:** Laura Matuschik, Gabriel Seifert, Katrin Lammich, Philipp Holzner, Yakup Tanriver, Stefan Fichtner-Feigl, Gerd Walz, Johanna Schneider, Bernd Jänigen

**Affiliations:** ^1^ Department of General and Visceral Surgery, Section of Transplant Surgery, Faculty of Medicine, Medical Center–University of Freiburg, Freiburg, Germany; ^2^ Department of Medicine IV, Faculty of Medicine, Medical Center–University of Freiburg, Freiburg, Germany

**Keywords:** ABO-incompatible kidney transplantation, complications, immunoadsorption, infections, mortality

## Abstract

ABO-incompatible (ABOi) living kidney transplantation (KTx) is an established procedure to address the demand for kidney transplants with outcomes comparable to ABO-compatible KTx. Desensitization involves the use of immunoadsorption (IA) to eliminate preformed antibodies against the allograft. This monocentric retrospective study compares single-use antigen-selective Glycosorb^®^ ABO columns to reusable non-antigen-specific Immunosorba^®^ immunoglobulin adsorption columns regarding postoperative infectious complications and outcome. It includes all 138 ABOi KTx performed at Freiburg Transplant Center from 2004–2020. We compare 81 patients desensitized using antigen-specific columns (sIA) to 57 patients who received IA using non-antigen-specific columns (nsIA). We describe distribution of infections, mortality and allograft survival in both groups and use Cox proportional hazards regression to test for the association of IA type with severe infections. Desensitization with nsIA tripled the risk of severe postoperative infections (adjusted HR 3.08, 95% CI: 1.3–8.1) compared to sIA. nsIA was associated with significantly more recurring (21.4% vs. 6.2%) and severe infections (28.6% vs. 8.6%), mostly in the form of urosepsis. A significantly higher proportion of patients with sIA suffered from allograft rejection (29.6% vs. 14.0%). However, allograft survival was comparable. nsIA is associated with a two-fold risk of developing a severe postoperative infection after ABOi KTx.

## Graphical Abstract

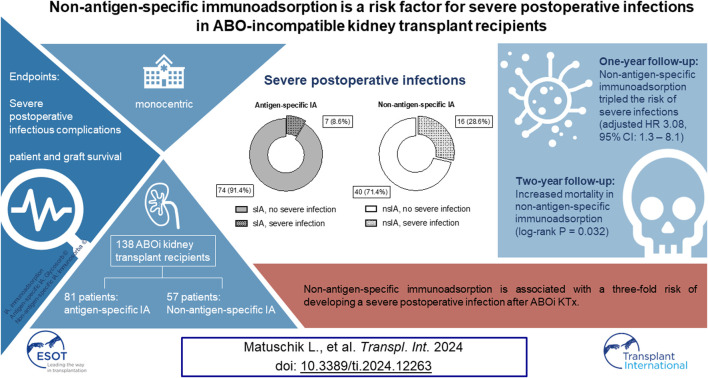

## Introduction

ABO-incompatible (ABOi) kidney transplantation (KTx) has become an established procedure to meet the demand for kidney transplants in patients with end-stage renal disease [1–3]. To prevent hyperacute or acute antibody-mediated allograft rejections due to pre-existing antibodies in the recipient, different ABOi protocols have evolved over the past years. These protocols have led to patient and graft survival rates comparable to conventional ABO-compatible (ABOc) transplantations [4, 5]. In accordance with Tydén’s initial desensitization protocol, our institution’s protocol has now been used for nearly 20 years [6]. It entails anti-CD20 treatment with Rituximab (375 mg/m^2^), serial immunoadsorption (IA) to eliminate preformed allograft antibodies and initiation of immunosuppressive maintenance therapy 9 days before the scheduled transplantation [1, 6]. From April 2004 until November 2011, antigen-specific Glycosorb^®^ ABO columns (sIA) were used to perform IA. These single-use columns contain the specific terminal carbohydrates of type A or B antigens as ligands linked to a sepharose matrix to eliminate donor-specific anti-A or anti-B IgM and IgG [7, 8]. From December 2011 until now, we have used reusable non-specific Immunosorba^®^ immunoglobulin adsorption columns (nsIA). They use staphylococcal antigen A, covalently linked to a sepharose matrix as the stationary phase of chromatography. Therefore, predominantly IgG1, IgG2 and IgG4, but also, to a lesser extent, IgA and IgM can be eliminated [9]. Several clinical studies found significantly higher rates of severe infections and infection-related mortality in ABOi transplanted patients compared to ABOc controls [10–12]. Only a small study investigated endpoint differences associated with IA modality in ABOi which showed no difference in infectious complications [13].

Based on our clinical experience, we suspected an association of nsIA with severe postoperative infectious complications.

To investigate this, we meticulously describe distribution of clinical covariates and infectious complications during the first postoperative year in nsIA and sIA KTx recipients. Secondly, we test whether nsIA is an independent risk factor for postoperative infections. Finally, we investigate whether nsIA is an independent predictor of recipient and graft survival in ABOi KTx.

## Patients and Methods

### Patients and Study Design

From 1 April 2004 until 16 June 2020 138 patients underwent ABOi living donor KTx in our Transplant Center. Mean patient follow-up is 7.4 years (2,703 days). This investigation is a monocentric retrospective analysis. The protocol was approved by our local IRB and registered in the German Clinical Trials Register (protocol number 296/20; registration number: DRKS00022385). All patients of the Freiburg living donor kidney program gave written informed consent for collecting and storing data in our living donor transplant registry.

The donor was examined during a 3-day inpatient stay. Statutory approval was given by the transplantation ethics committee of the District Medical Association Südbaden. The surgical procedure, graft preparation and recipient follow-up were performed as described before [14]. For the whole study period from 2004 to 2019, we used the ureteral stent OptiFlex 6 F 22 cm (OptiMed GmbH, Ettlingen, Germany). During the first two postoperative weeks, all recipients were treated on our transplant intermediate care ward.

Pre-transplant data were collected from the recipients’ local nephrologists and clinical data from clinical records. Clinical data were documented in the patients’ EMR during the whole study period. Follow-up data were documented in the EMR as well, as our nephrological transplant outpatient clinic uses the same hospital-wide electronic system. Delayed graft function was defined as the need of ≥1 dialysis treatments during the first 7 postoperative days. Graft loss was defined as the need to resume dialysis permanently caused by irreversible graft failure. Acute reversible graft failure was not included in statistics. The data of one patient, who died several hours after the transplantation due to myocardial infarction, are included in survival analysis, but not in the analysis of infections.

### Immunosuppression Regimes, Desensitization Protocol

Single-dose Rituximab (375 mg/m^2^ body surface; MabThera^®^, Roche Pharma AG, Grenzach-Wyhlen, Germany or Truxima^®^, Millmount Healthcare Ltd., Stamullen, Ireland) was administered approximately 30 days before the scheduled transplantation. Triple maintenance immunosuppression therapy was started 9 days before transplantation with the calcineurin-inhibitor tacrolimus (Prograf^®^, Astellas Seiyaku K.K., Tokyo, Japan; initial target trough level 12–15 ng/mL), mycophenolic acid (CellCept^®^, Roche Pharma AG, Grenzach-Wyhlen, Germany; 2,000 mg daily) and prednisone (30 mg daily). In case of tacrolimus intolerance (3 patients), the regimen was switched to cyclosporine (Sandimmun Optoral^®^, Novartis AG, Switzerland).

**FIGURE 1:**
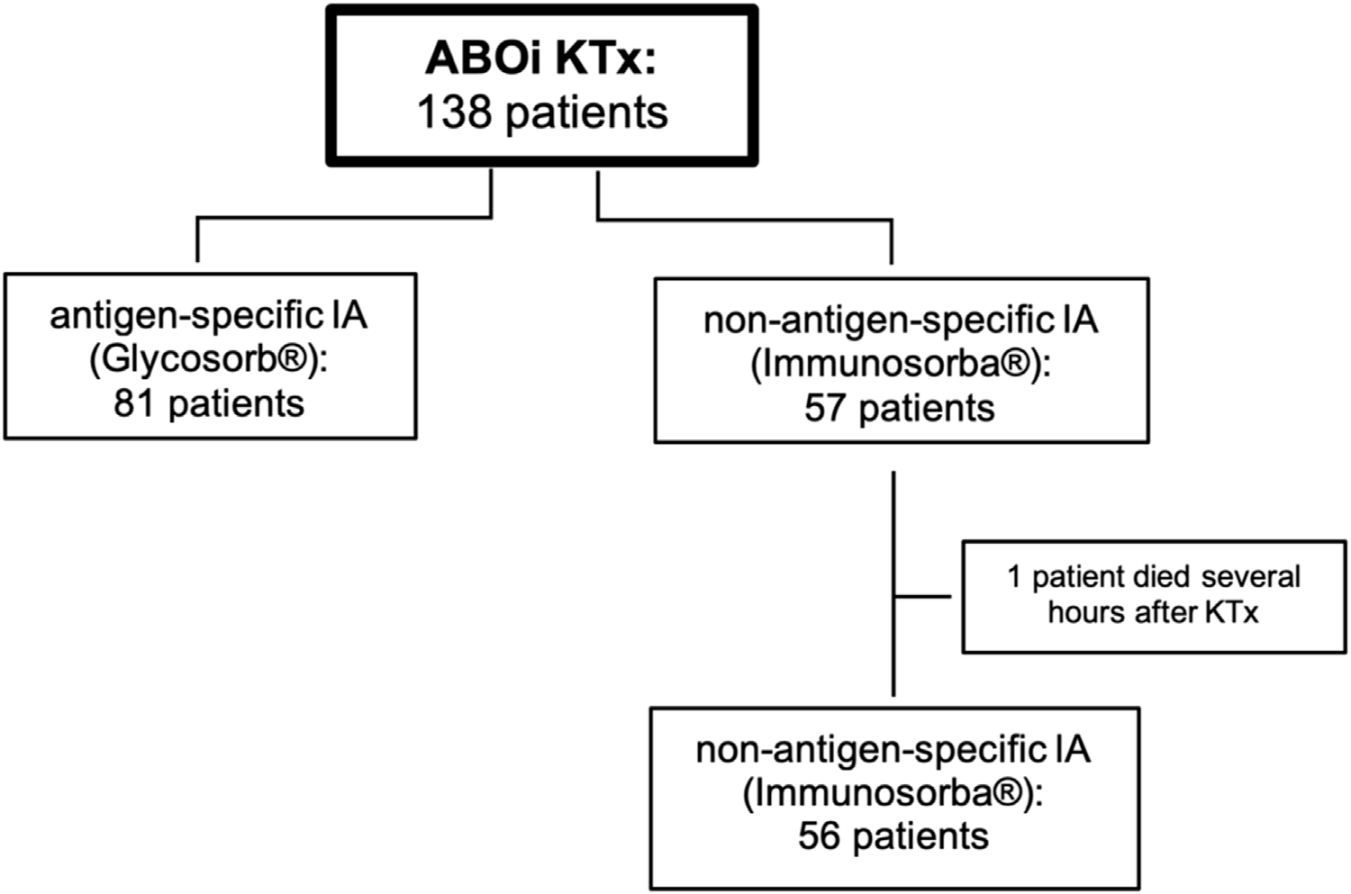
Study profile. 138 patients received ABO-incompatible living kidney transplantation after desensitization using different immunoadsorption columns. ABOi, ABO-incompatible; IA, immunoadsorption; KTx, kidney transplantation.

Additionally, induction therapy with 20 mg Basiliximab (Simulect^®^, Novartis AG, Basel, Switzerland) was administered on the day of transplantation and on day 4 after transplantation. In two patients, hypersensitivity against Basiliximab was detected; these patients received thymoglobulin (Sanofi-Aventis, Paris, France). IA was started 8 days before the scheduled transplantation date and performed on commercially available apheresis devices (Octo Nova™, Diamed Medizintechnik, Cologne, Germany) with hollow-fiber plasma separators (P2™, Fresenius Medical Care, Bad Homburg, Germany or Microplas MPS 07™, Bellco/Medtronic, Dublin, Ireland). The study profile is depicted in Figure 1. From April 2004 until November 2011, sIA was performed in 81 patients using antigen-specific Glycosorb^®^ ABO columns (Glycorex, Lund, Sweden). These single-use columns contain the specific terminal carbohydrates for A or B blood group antigens as ligands linked to a sepharose matrix to eliminate the specific anti-A or anti-B isoagglutinins. From December 2011 until June 2020, reusable non-antigen-specific Immunosorba^®^ immunoglobulin adsorption columns (Fresenius Medical Care, Bad Homburg, Germany) were used in 57 patients. These columns use staphylococcal antigen A, covalently linked to a sepharose matrix, as the stationary phase of chromatography. Immunoadsorption was performed every other day as described before [15], until the target titers of isoagglutinins (IgG and IgM) against donor erythrocytes were ≤1:4 on the day of surgery. If this target titer could not be reached until the scheduled date of KTx, IA was performed preoperatively on the day of surgery. In this case, the first titer measured after transplantation is used for statistics. Plasmapheresis (PPh) was initiated when isoagglutinin target titer levels could not be achieved by the preceding immunoadsorptions.

After transplantation, monitoring of isoagglutinin titers was performed daily during the first 7 days and every other day until the 14th postoperative day. If titers exceeded 1:8 IgM/IgG in the first week and 1:16 IgM/IgG in the second week post transplantation, immunoadsorption was scheduled on the same day.

IgG/IgM isohaemagglutinin titers were measured by our Medical Center’s Department of Transfusion Medicine. The first anti-donor isohaemagglutinin titers were quantified by a conventional tube centrifugation haemagglutination test (described by Winters et al. [16]). However, in mid-2007, i.e., after the first 20–30 ABOi KTx were performed in our center, a gel centrifugation haemagglutinin test with donor erythrocytes, able to distinguish between IgG and IgM isohaemagglutinins, was established (using the Diamed-Coombs-Anti-IgG^®^ and Diamed-ID-NaCl^®^ systems; DiaMed Diagnostika, Germany (current names: Coombs Anti-IgG and NaCl, BIO-RAD, Germany)). A detailed description of the method is provided by Wilpert et al. [15]. For quality control, the previous sample or pooled plasma of ten random donors are tested simultaneously. Antigen density proved to be stable, however, a direct correlation to renal tissue antigen density cannot be drawn.

Maintenance immunosuppression was administered as described before and not altered during the observation period [1]. All patients received anti-infective prophylaxis comprising valganciclovir for 100 days in CMV positive recipients and for 200 days in a high-risk constellation with a CMV seropositive donor, but negative recipient. Trimethoprim/sulfamethoxazole was administered for 6 months post transplantation and fluconazole prophylaxis until postoperative day 28.

### Infections

Risk of severe infectious complications during the first year after KTx was the primary endpoint of this retrospective study. In line with several other clinical studies [10, 12, 17], we distinguished between non-severe and severe infections in order to enhance the discriminatory power between uncomplicated postoperative developments and clinically relevant adverse events. Severe infections required the detection of pathogens in the blood stream or the state of sepsis according to the Third International Consensus Definitions for Sepsis and Septic Shock [18]. Every case of severe infection was objectified by the presence of organ dysfunction according to a SOFA (Sequential (Sepsis-related) Organ Failure Assessment) score ≥2 [18]. All cases of severe infections were treated within the University Medical Center in Freiburg. For all 138 patients, in-house and outpatient microbial findings (from the local nephrologists) were meticulously checked to determine infection severity in a valid way. We differentiated between bacteriuria, which included all urinary samples with detected pathogens, and urinary tract infections (UTIs) with a pathogen amount ≥ 10^5^/mL urine. Urine cultures were collected solely when an infection was suspected, i.e., when patients had symptoms such as dysuria, a urine test strip showing leukocyturia or nitrite-positivity, or the blood count showed elevated inflammatory values. Antibiotic prophylaxis to prevent UTI was not used in our center. Standard antibiotic treatment of UTIs had to be changed in 2019 following a “Rote Hand-Brief” of the European Medicines Agency and the German Institute for Drugs and Medical Devices, replacing norfloxacin with amoxicillin/clavulanic acid as standard antibiotic due to its potentially harmful side effects, especially in combination with corticosteroids. After obtaining the results of microbial urine culture, the antibiotic regimen was altered according to microbial resistance if necessary. Standard perioperative antibiotic prophylaxis comprised cefazoline and metronidazole. Recurring infections were defined as ≥ 2 infections (not necessarily of the same pathogen) during the first year after transplantation requiring therapy and/or hospitalization. Multi-drug resistance was defined according to the International Expert Proposal for Interim Standard Definitions for Acquired Resistance as “acquired non-susceptibility to at least one agent in three or more antimicrobial categories” [19]. Patients were considered CMV- or BKV-positive with virus replications over 1,000 IU/mL (serum) respectively. BKV nephropathy was defined as histologically proven BK virus infection. CMV was monitored once weekly via PCR during the initial hospitalization period after KTx. Afterwards, controls were made after 4, 8 and 12 weeks and after 3, 6, 9 and 12 months. From the second year after KTx, CMV was monitored based on clinical symptoms. Treatment of CMV infection included high-dose valganciclovir. Treatment was initiated in patients with virus replications over 1000 IU/mL and continued until replication rate was not detectable any more for two consecutive weeks. In cases of valganciclovir resistance, foscavir (and recently letermovir) was used. BKV PCR was performed after 3, 6, 9 and 12 months. When serum virus replications exceeded 1,000 IU/mL, a biopsy to rule out BKV nephropathy was conducted. If positive, immunosuppression was reduced, beginning by reducing the mycophenolate dose. Further adjustments of the immunosuppressive therapy are made stepwise, depending on the individual risk of rejection and BKV replication.

### Statistical Analysis

Results were defined as statistically significant when *p* < 0.05, all *p*-values being two-sided. All data were considered non-normal-distributed.

Severe postoperative infections were determined as primary endpoint. Categorical data are displayed as absolute and relative frequencies; a two-tailed Fisher’s Exact test was performed for comparison. Continuous data are expressed as median and 95% confidence interval (CI); for analysis, Mann-Whitney-U test was used. The cumulative incidence of postoperative infections was assessed by a competing risk analysis using the Aalen-Johansen estimate via the “survfit” function in R [R version 4.1.2 (2021-11-01) -- “Bird Hippie”], Gray’s test was added to test for a difference between groups over the entire follow-up period. To set the focus on the time of onset of infections, only the first episode of a non-severe and the first episode of a severe infection per patient were taken into account. Multivariable Cox proportional hazards regression analysis was performed to examine the association of IA modality and severe postoperative infections. “Severe infection” was modelled as a binary categorical outcome. “Severe infections” were distinguished from “non-severe infections” as defined above (bacteremia or SOFA score ≥2). The proportional hazard assumption was tested by visualizing Scaled Schoenfeld residuals vs. time (Supplementary Figure S1).

Patient and graft survival were investigated as secondary endpoints. To be able to include all 138 patients into this analysis, we set the cut-off at 2 years post transplantation. Cox proportional hazards regression analysis was performed to identify risk factors associated with graft loss and mortality during the first 2 years after ABOi KTx. Acute rejection episodes were not included in the multivariable models. We aimed at creating a regression model with adjustment for, according to our experience, clinically relevant and biologically plausible confounders. To validate the regression model used, Goodness-of-fit was examined via Partial likelihood ratio test, Wald test and Score test. Multicollinearity was evaluated using variance inflation factors (Supplementary Table S1).

GraphPad Prism version 9.3 (GraphPad Software, San Diego, CA, United States) and R version 4.1.2 (2021-11-01) -- “Bird Hippie were used to perform all statistical analyses and to visualize data.

## Results

### Baseline and Extended Characteristics

Postoperative infectious complications occurred in 99 cases during the first year after transplantation (72.2%). 23 patients (16.7%) developed a severe infection. Based on clinical experience, an increasing incidence rate of severe postoperative infectious complications was noted over the years.

Our cohort was split in two consecutive groups due to a switch in immunoadsorption column, the early group receiving sIA and the late group receiving nsIA (years 2004–2011 and 2011–2020). First, we investigated potential confounding demographic and clinical factors associated with these time windows.

Both IA groups were comparable concerning relevant donor and recipient characteristics, except for a significantly higher ASA category of nsIA patients (Table 1, for thorough analysis see Supplementary Tables S3, S4). Before desensitization, both groups had similar median isoagglutinin titers (1:16 (IgM), 1:64 (IgG), Supplementary Table S3, Supplementary Figure S2). In order to reach the target antibody titer of ≤1:4 before KTx, significantly more nsIA patients had to receive preoperative PPh (79% vs. 25%, Table 1. For a more detailed description of titer courses and IA treatments see Supplementary Table S3 and Supplementary Figure S2).

**TABLE 1:** Baseline and extended characteristics of donors and recipient groups receiving either antigen-specific or non-antigen specific immunoadsorption before ABO-incompatible kidney transplantation.

	Antigen-specific IA (81 patients)	Non-antigen specific IA (57 patients)
Recipients’ Characteristics		
Female sex, recipient (*n (%)*)	34 (42)	24 (42.1)
Age at transplantation, recipient (*years*)	46 (42, 49)	51 (45, 53)
BMI, recipient (*kg/m* ^*2*^)	24.3 (22.9, 25.5)	24.2 (23.3, 26.3)
ASA category, recipient (median (interquartile range))	3 (2, 3)	3 (3, 3)
Dialysis before transplantation (*n (%)*)	67 (82.72)	41 (71.93)
- Pre-emptive transplantation (*n (%)*)	14 (17.28)	16 (28.07)
Duration of dialysis before transplantation (*months*)	25 (17, 35)	17 (12, 27)
No. of HLA mismatches A + B + DR	4 (3, 4)	4 (3, 4)
PRA level (≥5%) (*n (%)*)	16 (19.75)	3 (5.26)
- Maximum PRA level	96%	66%
Donors’ Characteristics		
Female sex, donor (*n (%)*)	51 (62.96)	31 (54.39)
Age at transplantation, donor (*years*)	50 (48, 52)	53 (50, 55)
Genetic relationship (haploidentical parents or siblings) (*n (%)*)	27 (33.33)	17 (29.83)
Surgical Data		
Duration of surgery (*min*)	168 (150, 180)	146 (127, 157)
Ischemia time, total (*min*)	183 (172, 190)	172 (163, 185)
- Cold ischemia time	147 (136, 158)	142 (137, 157)
- Warm ischemia time	30 (29, 33)	25 (24, 28)
Duration of hospitalization (*days*)	19 (18, 21)	19 (18, 23)
Immunological Data		
Total no. of IA	5 (5, 6)	5 (4, 5)
- No. of preoperative IA	5 (5, 6)	4 (4, 5)
- IA on the day of surgery (*n (%)*)	53 (65.43)	13 (22.81)
No. of patients undergoing PPh (*n (%)*)	24 (29.6)	45 (78.95)
- Total no. of PPh when needed	2.5 (2, 3)	2 (2, 3)
- No. of preoperative PPh when needed	2.5 (2, 3)	2 (2, 2)
- PPh on the day of surgery (*n (%)*)	2 (2.47)	7 (12.28)
High IgM/IgG titer (≥1:256) before Rituximab (*n (%)*)	22 (27.85)	14 (24.56)
- Total no. of IA	7 (5, 9)	5 (4, 6)
- No. of patients undergoing PPh (*n (%)*)	10 (45.46)	14 (100)
- Total no. of PPh when needed	3 (1, 5)	2 (2, 5)
- Total no. of IA + PPh	9.5 (8, 17)	8.5 (7, 13)
Total no. of IA + PPh (all patients)	6 (5, 8)	6 (6, 7)
Infectious Complications		
Any infection (*n (%)*)	59 (72.8)	40 (71.4)
- Days from KTx to first infection	11 (9, 20)	9 (7, 12)
Recurring infections (*n (%)*)	5 (6.17)	12 (21.43)
Severe infection (*n (%)*)	7 (8.6)	16 (28.6)
- Days from KTx to first sepsis	61 (5, 239)	56 (22, 126)
- Septic shock (*n (%)*)	2 (28.6)	5 (31.3)
Bacterial and Opportunistic Infections		
Blood culture pathogen detection (*n (%)*)	7 (8.6)	16 (28.6)
Urinary tract infections (*n (%)*)	38 (46.9)	24 (42.9)
- Multidrug-resistant bacteria (*n (%)*)	8 (21.1)	8 (33.3)
- Urosepsis (*n (%)*)	4 (10.5)	13 (54.2)
- Duration of ureteral stenting (*days*)	14 (13, 14)	20 (13, 40)
Viral Infections		
BKV		
- BK viremia (*n (%)*)	10 (12.4)	16 (287)
- Highest BK virus replication no. (*copies/mL*)	124,340 (14,100, 1,341,500)	138,864 (30,000, 86,300)
- Days from KTx to first BKV positivity	161.5 (85, 289)	100 (63, 180)
- Duration of BK viremia (*days*)	163 (93, 355)	283 (216, 567)
- BK virus nephropathy (in BKV + patients) (*n (%)*)	4 (40)	4 (26.67)
CMV		
- CMV status of donor positive (*n (%)*)	46 (56.8)	27 (47.4)
- CMV status of recipient negative (*n (%)*)	36 (44.4)	28 (49.1)
- Risk constellation (D +/R -) (*n (%)*)	16 (19.8)	9 (15.8)
- CMV viremia (*n (%)*)	4 (4.9)	4 (7.0)
- Highest CMV replication no. (*copies/mL*)	3,895 (3,200, 5,250)	12,000 (2,000, 732,000)
- Days from KTx to first CMV positivity	34.5 (13, 234)	179.5 (118, 570)
- Duration of CMV viremia	18 (6, 606)	60.5 (13, 572)

Median values are provided (95% CI, of median) unless indicated otherwise. American Society of Anesthesiologists (ASA) category is shown in median (interquartile range). BKV: BK, virus; CMV: cytomegalovirus; D: donor; IA: immunoadsorption; IA: immunoadsorption; IgG: immunoglobulin G; IgM, immunoglobulin M; KTx: kidney transplantation; PPh: plasmapheresis; R: recipient. Patients were considered CMV- or BKV-positive with virus replications over 1000 IU/mL (serum) respectively.

### Infectious Complications

**FIGURE 2:**
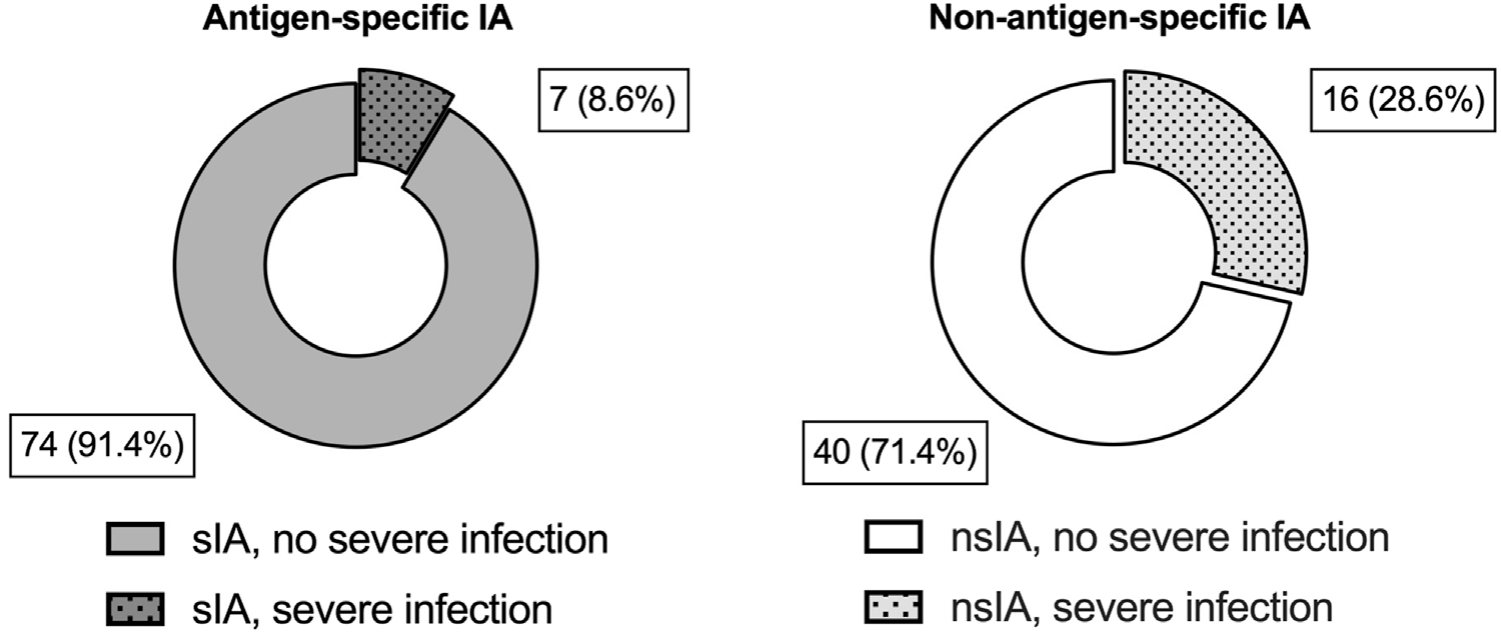
Severe infectious complications during the first year after ABO-incompatible kidney transplantation. Shown are absolute numbers and percentages for each IA group for severe infectious complications during the first year after ABOi KTx. Grey blocks indicate patients desensitized with antigen-specific IA (sIA); white blocks represent patients receiving non-antigen-specific IA (nsIA). The incidence of severe infections was compared using a two-tailed Fisher’s exact test (sIA vs. nsIA: 7 (8.6%) vs. 16 (28.6%), *p* = 0.004).

Postoperative infections occurred frequently in both IA groups with a slightly higher incidence in sIA (Table 1). The crude risk for severe infections and septic shock was higher in nsIA (28.6% vs. 8.6%; 21.7% vs. 12.5%, Table 1; Figure 2).

**FIGURE 3:**
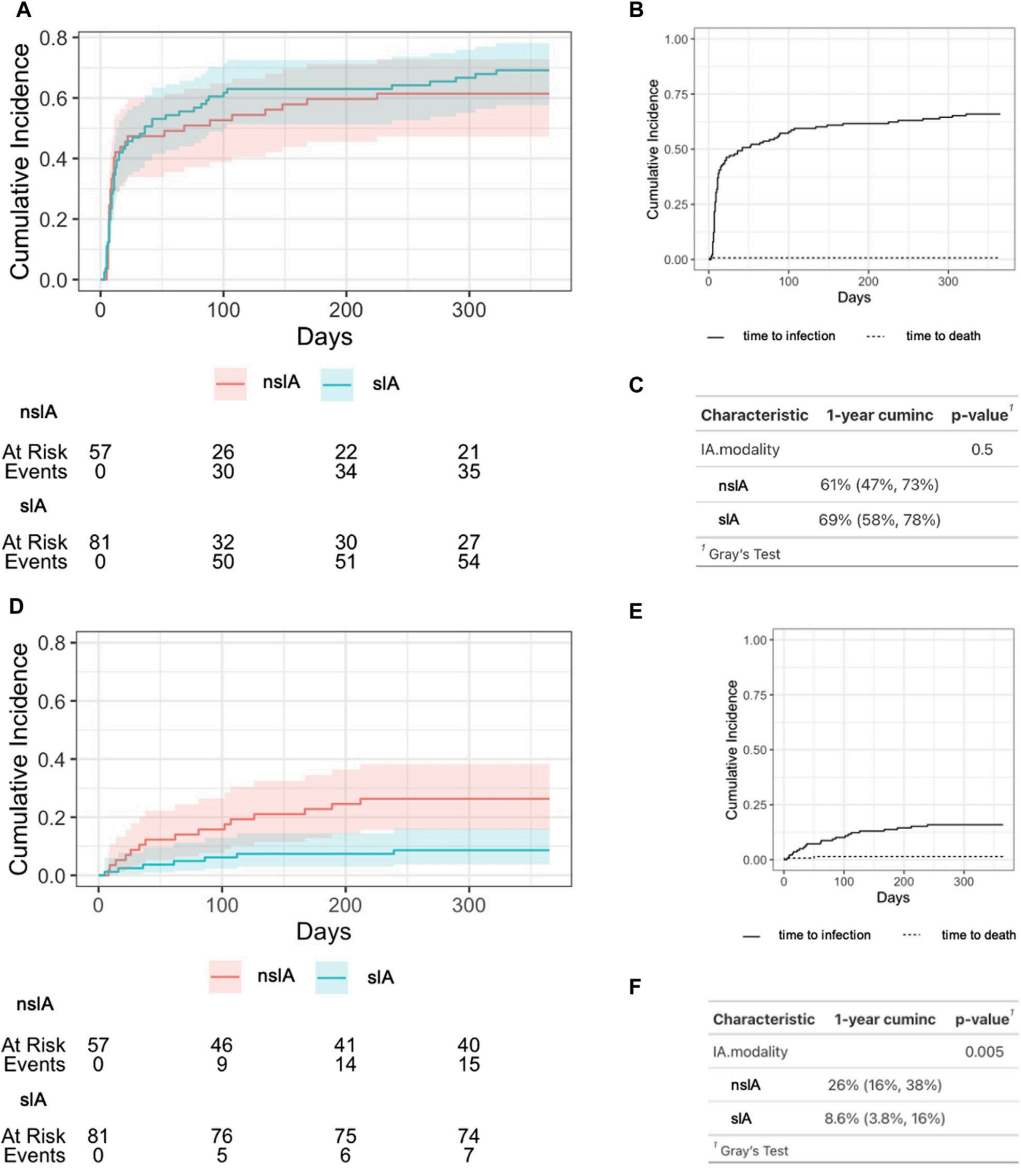
Cumulative incidence of infectious complications after ABO-incompatible kidney transplantation. **(A)** Time period until the occurrence of the first infection after transplantation according to IA modality. nsIA: non-antigen-specific immunoadsorption; sIA: antigen-specific immunoadsorption, **(B)** Cumulative incidence of any infectious complication after KTx for the whole cohort. **(C)** Estimation of 1-year cumulative incidence of any post-transplant infection. “cuminc”: cumulative incidence. **(D)** Time period until the occurrence of the first severe infection after KTx according to IA modality. nsIA: non-antigen-specific immunoadsorption; sIA, antigen-specific immunoadsorption, **(E)** Cumulative incidence of severe infectious complications after KTx for the whole cohort. **(F)** Estimation of 1-year cumulative incidence of severe post-transplant infections. A severe infection required the detection of pathogens in the blood stream or a SOFA score ≥2. To estimate the cumulative cause-specific infection-free survival, the Aalen-Johansen estimate was used; Gray’s test was then used to test for a difference between cause-specific survival functions.

These findings are congruent with a competing risk analysis comparing the cumulative incidence of non-severe vs. severe infections in sIA and nsIA (Figure 3). In both groups, esp. uncomplicated urinary tract infections (UTIs), were common during the first 3 months after KTx, affecting over 50% of all patients (Figure 3A). In the sIA group, 91.5% of the depicted first episode of a postoperative non-severe infection were UTIs, with only 5 cases of other foci (3 x pulmonary, 1 BKV nephropathy and 1 case with unclear focus). Similarly, 90% of the first non-severe infections in the nsIA group were UTIs, with only two further cases of pneumonia, 1 catheter sepsis and 1 BKV nephropathy.

By contrast, the median onset of the first severe infection after KTx was after 2–3 months (Table 1; Figure 3D). Within the first year after KTx, the incidence of severe infections was significantly higher in nsIA compared to sIA (Figure 3F). Recurring infections were significantly more frequent in nsIA (21.4% vs. 6.2%, Table 1).

**FIGURE 4:**
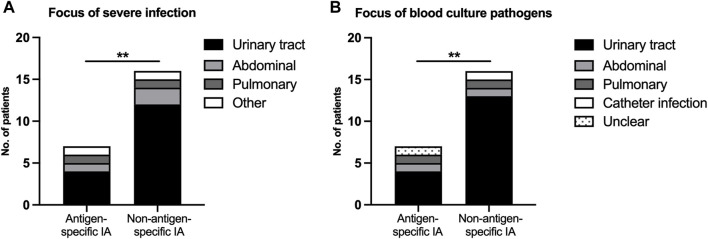
Severe infectious complications after ABO-incompatible kidney transplantation. **(A)** Focus of severe infection, **(B)** Focus of blood culture pathogens. A severe infection was defined as the detection of pathogens in the blood stream or a SOFA score ≥2. *p*-values are estimated with a two-tailed Fisher’s exact test. *Denotes statistical significance between antigen-specific IA and non-antigen-specific IA (**p < 0.01).

**FIGURE 5:**
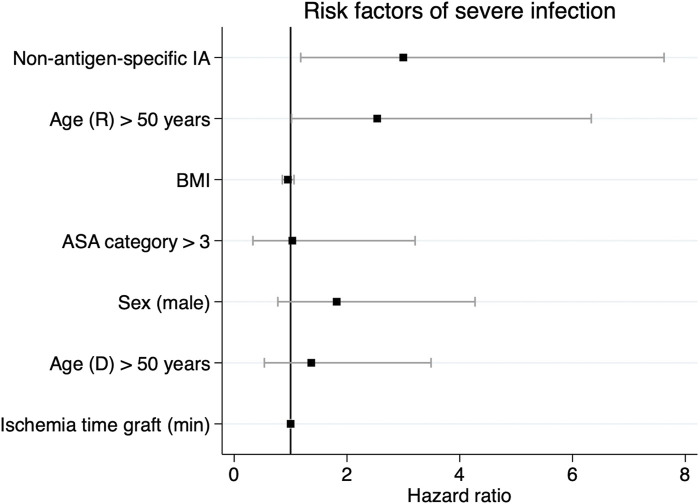
Regression coefficient plot visualizing the relative hazard of postoperative severe infections during the first year after ABO-incompatible kidney transplantation. Provided are parameter estimates with 95% confidence limits. IA, ASA category, age >50 years and recipient sex were included as categorical variables. ASA, American Association of Anesthesiologists; IA, immunoadsorption.

In accordance with a higher risk of severe infections, bacteremia was more common in nsIA (28.6% vs. 8.6%, Table 1), the predominant focus in both groups being the urinary tract (Figure 4, Supplementary Table S2). The time of onset for UTIs (40 days vs. 41 days post-transplant) as well as the proportion of patients suffering from an uncomplicated UTI (sIA: 46.9% vs. nsIA: 42.9%) was similar between the IA groups. Urosepticemias, however, made up to 54.2% of all UTIs in nsIA, compared to 10.5% in sIA (Table 1). The spectrum of detected pathogens included slightly more multi-drug resistant bacteria in nsIA (Table 1; Figure 5; for antibiotic susceptibility profiles of MDR pathogens see Supplementary Table S5). Concerning severe infections, the predominant pathogen in both groups was *Escherichia coli*, with a higher percentage of *Escherichia coli with extended-spectrum beta-lactamases* (ESBLs) in the sIA group (35% vs. 20%). Whereas *Enterococcus faecalis* was the second most detected pathogen causing UTIs of patients receiving sIA, it was *Klebsiella species* in the nsIA group (Supplementary Figure S3).

Septic shock occurred in 2 cases, i.e., 28.6% of severe infections, in the sIA group. One patient developed *C. difficile*-associated colitis, in the second patient, the focus remained unclear. In the nsIA group, five patients suffered from septic shock, equivalent to 31.3% of severe infections in this group. The underlying causes were two cases of urosepsis with multi-organ failure (*K. pneumoniae, E. coli*), one abdominal sepsis due to caecal ischemia with consecutive perforation (*E. faecium VRE*), an abdominal wall phlegmon with development of an abscess (*E. coli*) and a *parainfluenza-2* viral pneumonia.

As the main increase of severe infections in the nsIA group was due to urosepsis, other UTI-associated factors were analyzed: whereas the manufacturer and product of the ureteral stents did not change, the duration of stenting was significantly longer in the nsIA group (20 (13, 40) days vs. 14 (13, 14) days in the sIA group, Table 1). Additionally, when comparing only patients that needed additional PPh to their IA treatment, we found significantly more cases of urosepsis within the nsIA + PPh group (11 cases) than within the sIA + PPh group (only one case).

BK viremia was detected significantly more often in nsIA (28.6% vs. 12.4%, Table 1) and duration of viremia was significantly longer (283 vs. 163 days, Table 1). Unadjusted comparison of onset of BK viremia, highest BKV replication numbers and BKV nephropathy were comparable (Table 1). No differences regarding CMV infections were detected (Table 1).

To further investigate the suspected association of nsIA and severe postoperative infections, firstly, we performed a more thorough analysis of the 23 patients (16.7%) that developed a severe infection (Table 2). In the first group, average age was higher, length of hospitalization was longer and the proportion of female recipients was higher. There was no difference regarding BMI, ASA category, the number of mismatches, duration of surgery and ischemia time. 69.6% of patients with severe infections received nsIA. To take PPh as immunomodulating factor into account, we performed several subgroup analyses, e.g., of patients who exclusively received IA, but no PPh. Of 57 patients (70.4%) in the sIA group, 2 (3.5%) suffered from a severe infection. Only 12 patients (21.1%) in the nsIA group did not require PPh. However, 3 (25%) developed a severe infection. In this small, crude subgroup analysis, nsIA is associated with a higher risk of severe infections (Table 2).

**TABLE 2:** Characteristics of patients with and without severe infectious complications during the first year after ABO-incompatible kidney transplantation.

	Severe postoperative infection (23 patients, 16.7%)	No severe postoperative infection (114 patients, 82.6%)
Recipients’ Characteristics		
Female sex, recipient (*n (%)*)	13 (56.5)	44 (38.6)
Age at transplantation, recipient (*years*)	55 (45, 59)	47 (44, 49)
BMI, recipient (*kg/m* ^*2*^)	24.9 (20.2, 26.7)	24.3 (23.3, 25.5)
ASA category, recipient	3 (3, 3)	3 (3, 3)
Dialysis before transplantation (*n (%)*)	15 (65.2)	93 (81.6)
Duration of dialysis before transplantation (*months*)	10 (0, 27)	15.5 (10, 20)
No. of HLA mismatches A + B + DR	4 (3, 5)	4 (3, 4)
- A mismatch	1 (1, 2)	1 (1, 1)
- B mismatch	2 (1, 2)	1 (1, 2)
- DR mismatch	1 (1, 1)	1 (1, 1)
PRA level (≥5%) (*n (%)*)	4 (17.4)	15 (13.2)
Surgical data		
Duration of surgery (*min*)	148 (118, 157)	158.5 (146, 172)
Ischemia time, total (*min*)	166 (156, 193)	177.5 (172, 187)
- Cold ischemia time	140 (135, 161)	145.5 (138, 156)
- Warm ischemia time	25 (22, 30)	39.5 (27, 31)
Duration of hospitalization (*days*)	23 (17, 29)	19 (18, 20)
Immunological Data		
Non-antigen-specific immunoadsorption (*n (%)*)	16 (69.6)	40 (35.1)
Patients with high IgM/IgG titer (≥1:256) before Rituximab (*n (%)*)	5 (21.7)	31 (27.2)
Total no. of IA	5 (4, 6)	5 (5, 5)
No. of patients undergoing PPh (*n (%)*)	15 (65.2)	53 (46.5)
Total no. of IA + PPh (all patients)	6 (6, 7)	6 (5, 7)
Subgroups based on desensitization (*n (%)*)		
- Patients that only received sIA (57 of 81 pat.)	2 (3.5)	55 (96.5)
- Patients that only received nsIA (12 of 57 pat.)	3 (25)	9 (75)
- Patients with sIA + PPh (24 of 81 pat.)	2 (8.3)	22 (91.7)
- Patients with nsIA + PPh (45 of 57 pat.)	13 (28.9)	32 (71.1)

Median values are provided (95% CI, of median) unless indicated otherwise. ASA, american society of anesthesiologists; BMI, body mass index; HLA, human leukocyte antigen; IA, immunoadsorption; IgG, immunoglobulin G; IgM, immunoglobulin M; PPh, plasmapheresis; PRA, panel-reactive antibody.

Secondly, we aimed to identify independent risk factors of severe infectious complications after ABOi KTx using a multivariable Cox regression analysis. Clinically relevant confounders such as age, ASA category, sex and ischemia time were included in the model. NsIA was independently associated with a 3.08 HR with severe infections (95% CI: 1.3–8.1, Table 3; Figure 5). Moreover, recipient age >50 years was associated with severe infections (HR 2.53, 95% CI: 1.0–6.0, Table 3; Figure 5).

**TABLE 3:** Relative hazard of postoperative severe infections during the first year after ABOi KTx by risk factors.

Predictor variable	HR	95% CI	*p*-value
Non-antigen-specific IA	3.083	1.3–8.1	0.015
Age (R) > 50 years	2.534	1.0–6.6	0.045
BMI	0.954	0.8–1.1	0.410
ASA category >3	0.805	0.1–3.0	0.727
Sex (male)	1.797	0.8–4.4	0.210
Age (D) > 50 years	1.386	0.6–3.7	0.215
Ischemia time graft (min)	1.000	0.99–1.0	0.931

IA, ASA, category, age >50 years and recipient sex were included as categorical variables. Goodness-of-fit tests and VIFs, are provided as Supplementary Material (Supplementary Table S1). ASA, american association of anesthesiologists; CI, confidence interval; D, donor; HR, hazard ratio; IA, immunoadsorption; R, recipient.

### Graft Function and Patient Survival

After identifying nsIA as an independent risk of severe infections, we aimed at analyzing its potential impact on graft function and patient survival.

Graft function was equal in both IA groups, and delayed graft function, creatinine levels at discharge and at last follow-up were comparable (Supplementary Table S6). A significantly higher number of sIA patients developed any type of graft rejection (29.6% vs. 14.0%) requiring significantly more graft biopsies (Supplementary Table S6; for a detailed description of rejection episodes and their individual treatment see Supplementary Table S7).

During a follow-up period of 2 years, five cases of graft loss were recorded in total (Supplementary Table S6). In sIA, two (66.7%) graft losses occurred due to chronic rejection, while thrombosis was the cause of the third graft loss. By contrast, both graft losses in nsIA were caused by urosepsis, in one case accompanied by coagulopathy and hemorrhagic shock (Supplementary Table S6; for description of individual etiologic factors of graft failures see Supplementary Table S8).

To determine independent risk factors associated with graft loss during the first 2 years after KTx, a Cox multivariable regression analysis was conducted (Table 4). IA modality was no independent risk for graft loss (Table 4); however, it was independently associated with recipient age >50 years (HR 5.14, 95% CI: 1.2–32.6), female sex (HR 6.38, 95% CI: 1.6–38.3) and warm ischemia time (HR 1.006 per minute, 95% CI: 1.00–1.01).

**TABLE 4:** Relative hazard of graft loss during the first 2 years after transplantation by risk factors.

Predictor variable	HR	95% CI	*p*-value
Non-antigen-specific IA	2.790	0.75–12.41	0.137
Age (R) > 50 years	5.142	1.22–32.56	0.042
BMI	1.113	0.96–1.29	0.150
ASA category >3	0.290	0.01–3.27	0.391
Sex (female)	6.382	1.59–38.26	0.018
Age (D) > 50 years	1.292	0.30–7.64	0.746
Ischemia time graft (min)	1.006	1.00–1.01	0.007

IA, ASA, category, recipient sex and age >50 years were included as categorical variables. ASA, american association of anesthesiologists; BMI, body mass index; CI, confidence interval; D, donor; HR, hazard ratio; IA, immunoadsorption; R, recipient.

**FIGURE 6:**
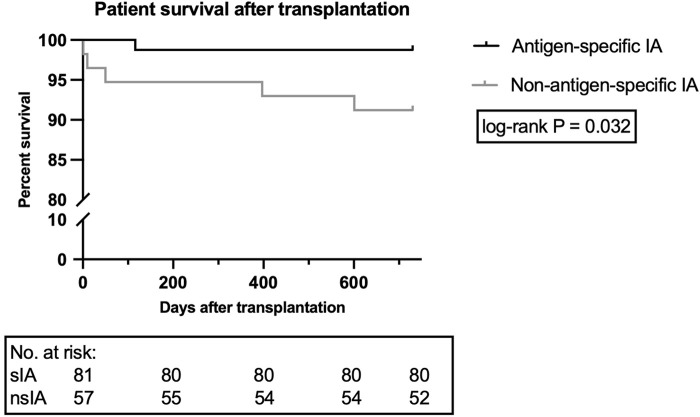
Patient survival during the first 2 years after ABOi kidney transplantation. Data are shown for 81 patients receiving sIA and 57 patients receiving nsIA. To display the time period until the occurrence of patient death during the first 2 years after kidney transplantation via Kaplan-Meier graph, a log-rank test was utilized. nsIA, non-antigen-specific immunoadsorption; sIA, antigen-specific immunoadsorption.

Despite a similar number of deaths during the whole follow-up period (mean: 7.4 years) and a low overall mortality rate, a higher number of nsIA patients died during the first 2 years (8.8% vs. 1.2%, Supplementary Table S6; log-rank *p* = 0.032; Figure 6). In sIA, three patients died from sepsis-related multi-organ failure–only one of them during the first 2 years after KTx–one patient died due to metastatic squamous cell carcinoma, and the cause of one death remains unknown. In nsIA, all recorded deaths occurred during the first 2 years after KTx: two patients died from septic multi-organ failure, two patients due to cardiogenic shock and one patient due to metastatic lung carcinoma (see Supplementary Table S9 for causes of death with functioning graft).

To identify mortality-associated risk factors, a Cox proportional hazards regression analysis was implemented. Univariable analysis revealed an increased mortality risk for nsIA patients (HR: 7.4, 95% CI: 1.2–140.9) as well as for recipients >50 years (HR: 7.5, 95% CI: 1.2–143.8, Table 5).

**TABLE 5:** Relative hazard of patient death during the first 2 years after transplantation by risk factors.

	Univariable			Multivariable		
Predictor variable	HR	95% CI	*p*-value	HR	95% CI	*p*-value
Non-antigen-specific IA	7.359	1.2–140.9	0.069	6.203	0.91–131.0	0.110
Age (R) > 50 years	7.511	1.2–143.8	0.066	7.954	1.09–169.9	0.077
BMI	1.043	0.8–1.2	0.659	1.006	0.82–1.22	0.951
ASA category >3	2.466	0.1–15.3	0.410	1.629	0.06–15.63	0.712
Sex (female)	2.973	0.6–21.4	0.208	3.645	0.67–28.53	0.152
Age (D) > 50 years	1.671	0.3–12.1	0.553	0.701	0.11–5.80	0.713
Ischemia time graft (min)	0.999	0.98–1.01	0.920	1.003	0.98–1.01	0.595

Univariable and multiple Cox proportional hazards regression. IA, ASA, category, recipient sex and age >50 years were included as categorical variables. ASA, american association of anesthesiologists; BMI, body mass index; CI, confidence interval; D, donor; HR, hazard ratio; IA, immunoadsorption; R, recipient.

After adjusting for age, BMI, ASA category, sex and ischemia time, only recipient age >50 years was independently associated with two-year mortality (HR: 7.95, 95% CI: 1.09–169.9, Table 5).

## Discussion

Based on our clinical experience, we had hypothesized nsIA to be associated with severe postoperative infectious complications. Indeed, in this cohort, IA modality was independently associated with risk of severe infections and an increased two-year mortality.

ABOi KTx has become a well-established method to expand the living donor pool with patient and graft survival similar to ABOc KTx. However, it is associated with higher postoperative infectious complication risk [4, 10, 11, 17, 20]. Intensified immunosuppression protocols contribute to impaired pathogen defense. Immunoadsorption is among the established methods to reduce the recipient’s level of preformed anti-A/B isoagglutinins against the allograft [21]. Existing protocols differ regarding the selectivity of antibody removal: antigen-specific immunoadsorption was implemented in 2003, soon to be followed by non-antigen-specific IA protocols [6, 22].

So far, the impact of different IA protocols concerning overall patient survival and graft function was mostly compared to ABOc cohorts [3, 4, 10, 15]. Studies from London and Heidelberg found a significant rise in death rates in ABOi due to infectious complications during the early posttransplantation period [4, 17, 23]. They reported mainly opportunistic and viral infections, indicating an increased immunosuppressive burden in ABOi compared to ABOc KTx [17, 24]. In contrast to this, we did not find increased infection rates in ABOi patients compared to ABOc in our center [1].

In 2012, we transitioned from sIA (modified Swedish protocol) [1, 15] to nsIA due to the substantial economic burden of blood group-specific single use columns (∼5 IA/patient). Morath et al. and others had not found any differences in graft function and patient survival using nsIA [22, 25]. Thölking et al. compared the same IA columns as used at our Transplantation Center, namely, the antigen-specific Glycosorb^®^ column to the non-antigen-specific Immunosorba^®^ column [13]. An association of postoperative bacterial and viral infections with IA modality was not found [13, 22]. In our substantially bigger cohort, nsIA is independently associated with a two-fold risk of severe postoperative infections, mainly occurring during the first 6 months postoperatively. This is similar to the multi-center analysis conducted by Opelz et al., who found an increase in infections during the first year after ABOi KTx [4]. The risk of early postoperative infections may be associated with Rituximab administration 30 days prior to the scheduled KTx (Swedish protocol) [26]. Rituximab associated B-cell depletion was found to last for almost half a year [27]. During this time, an additional hypogammaglobulinemia was observed, hence enhancing the risk of infections [28]. This risk predisposition appears to be significantly increased in nsIA patients.

The main increase of severe infections in the nsIA group was due to urosepsis. The urosepsis rate of our nsIA group, however, is similar to published data from other centers that desensitized their KTx recipients by non-antigen-specific immunoadsorption [10]. Potential factors other than IA, which could be associated with the development of postoperative urosepsis in the nsIA group, are the longer duration of ureteral stenting and additionally, the need for PPh on top of IA treatment.

The risk of BKV positivity was significantly higher and the duration of BK viremia significantly longer in nsIA. Data on the duration of BK viremia after semi-selective IA are scarce. Significantly higher incidences of BKV nephropathy in ABOi recipients desensitized by sIA or PPh were found in two studies from the United Kingdom and United States in comparison to ABOc recipients and HLA-incompatible patients, respectively [29, 30]. Speer et al. found a higher risk of BKV positivity specifically in “high-titer” compared to “low-titer” patients within their ABOi cohort [10]. In line with other groups with comparable immunosuppressive regimens and desensitization protocols, we did not find any differences concerning CMV positivity [10, 13, 22].

Although the overall mortality rate was low, retrospective analysis found an increased mortality during the first 2 years after KTx in nsIA patients. This finding has not been reported previously [13, 22]. Most recent studies compare ABOc to ABOi patients and show conflicting results. Whereas Genberg et al. did not report any differences in patient survival, others found a significant rise in death rates due to infectious complications during the early post-transplant period in ABOi patients [3, 4, 17, 23]. In line with our retrospective analysis, univariable Cox proportional hazard regression revealed an elevated mortality risk during the first 2 years post-transplantation in nsIA compared to sIA. However, after adjusting for clinical confounders, the Cox regression model did not show an independent effect of the IA column on mortality. Mortality was <5% during the first 2 years post transplantation in our cohort. The low number of adverse events, as well as the large confidence interval, indicate a lack of statistical power. Our data, combined with our clinical experience, strongly suggest that there is a relevant difference in risk of mortality. However, pooled analysis of larger data is necessary to make a valid inference.

The risk of severe infections in our cohort is higher than in other studies. This may be due to a stricter preoperative desensitization protocol. We aimed at preoperative IgM and IgG isoagglutinin target titers ≤1:4, whereas the Stockholm protocol accepts ≤1:8 and other studies used ≤1:16 as cut-off pre-transplantation. A titer of >1:16 has been associated with an elevated risk of antibody mediated rejections [10, 13, 22, 26, 31]. Interestingly, there is increasing evidence of successful ABOi KTx without preoperative anti-blood group antibody removal in patients with low initial titers, even in pediatric patients, with comparable outcomes as following ABOc KTx [17, 32, 33].

The total number of preoperative IA and PPh treatments did not differ between the two groups and was similar to Thölking et al. [13]. Significantly more nsIA patients had to receive preoperative PPh, mostly due to limited adsorption of IgM isoagglutinin by IA [34]. This may augment the predisposition to postoperative infections due to the non-selective depletion of antibodies by apheresis [35, 36]. Therefore, postoperative IA treatments were scheduled if titers exceeded 1:8 during the first 7 days and 1:16 during the following week. This displays a stricter strategy than performed by other groups and resulted in lower rates of allograft rejections compared to their cohorts [10, 22].

Although currently 80% of our ABOi patients need preoperative PPh, we prefer non-antigen-specific IA with intercurrent PPh when needed compared to a desensitization protocol solely based on PPh. PPh is accompanied by an alteration of coagulation (when exchanged with human albumin), which may lead to more frequent postoperative bleeds. Alternatively, exchanging the patient’s plasma volume with fresh frozen plasma (FFP), is associated with exposure to unwanted allogenic components of FFPs as well as the transfusion-related risk of infection [37].

Immunoadsorption, on the contrary, allows the elimination of isoagglutinins and only slightly changes the concentration of coagulation factors and other immunoglobulins.

Although this analysis currently represents the largest patient collective comparing sIA to nsIA, its limitations include the observational monocentric approach and a cohort consisting mainly of patients of European origin. Data were collected retrospectively over a large time frame (16 years) and not contemporaneously, which makes biases inherent. Due to our team’s increasing clinical experience and protocol standardization, the group that received nsIA comprised significantly more patients suffering from pre-existing medical conditions (higher ASA category). This may confound the association between IA modality and infectious complications. In this respect, a prospective randomized and at least one-side-blinded comparison of IA columns should be conducted. Especially regarding survival analysis, larger patient cohorts are needed to reduce the risk of inconclusive results.

This is the first study to show that nsIA in ABOi KTx is an independent risk for severe postoperative infectious complications. sIA correlates with increased rejection rates, however, with a similar long-term graft survival.

## Data Availability

The original contributions presented in the study are included in the article/Supplementary Material, further inquiries can be directed to the corresponding author.
