# Supplementary material for: Non-antigen-specific Immunoadsorption Is a Risk Factor for Severe Postoperative Infections in ABO-Incompatible Kidney Transplant Recipients
Source: Transpl Int. 2024 Mar 14;37:12263. doi: 10.3389/ti.2024.12263 (PMC10974667; doi:10.3389/ti.2024.12263)
Supplement: Supplementary file 1 [file DataSheet1.docx]

**Supplemental Digital Content:**

**Figure S1.** **The proportional hazard assumption of the utilized cox multivariate regression models was tested by visualizing Scaled Schoenfeld residuals vs. time.** **A:** Severe infections. **B:** Graft loss. **C:** Patient survival.

**Table S1.** **Relative hazard of postoperative severe infections during the first year after transplantation by risk factors.** In all cox regression models (Tables 2, 5, 6), multicollinearity was checked using variance inflation factors which were < 10. IA, ASA category, age > 50 years and recipient sex were included as categorical variables. ASA: American Association of Anesthesiologists; BMI: body mass index; CI: confidence interval; D: donor; HR: hazard ratio; IA: immunoadsorption; R: recipient.

| **PREDICTOR VARIABLE** | **HR** | **95% CI** | **P-value** | **VIF** |
| --- | --- | --- | --- | --- |
| Non-antigen-specific IA | 3.083 | 1.3 – 8.1 | 0.015 | 1.80 |
| Age (R) > 50 years | 2.534 | 1.0 – 6.6 | 0.045 | 1.91 |
| BMI | 0.954 | 0.8 – 1.1 | 0.410 | 8.68 |
| ASA category > 3 | 0.805 | 0.1 – 3.0 | 0.727 | 1.15 |
| Sex (male) | 1.797 | 0.8 – 4.4 | 0.210 | 1.71 |
| Age (D) > 50 years | 1.386 | 0.6 – 3.7 | 0.215 | 2.61 |
| Ischemia time graft (min) | 1.000 | 0.99 – 1.00 | 0.931 | 5.52 |

**Table S2. Infectious complications after ABO-incompatible kidney transplantation.** IA: immunoadsorption; IgG: immunoglobulin G; IgM: immunoglobulin M. All data are shown in n (%).

| **INFECTIOUS COMPLICATIONS** | **Antigen-specific IA** | **Non-antigen specific IA** |
| --- | --- | --- |
| **Focus of severe infection *(n (%))*** |  |  |
| - Abdominal | 1 (14.3) | 2 (12.5) |
| - Urinary tract | 4 (57.1) | 12 (75) |
| - Pulmonary | 1 (14.3) | 1 (6.3) |
| - Other | 1 (14.3) | 1 (6.3) |
| **Patients with high IgM/IgG titer (≥ 1:256) before Rituximab *(n (%))*** | 22 (27.85) | 14 (24.56) |
| - Severe infections after KTx | 1 (4.55) | 4 (28.57) |
| - Recurring infections after KTx | 2 (8.33) | 6 (42.86) |
| - BK viremia | 4 (18.18) | 4 (28.57) |
| - CMV viremia | 1 (4.55) | 3 (21.43) |
| **Blood culture pathogen detection *(n (%))*** | 7 (8.64) | 16 (28.57) |
| - Multidrug-resistant bacteria *(n (%))* | 1 (14.29) | 2 (12.5) |
| - Cause *(n (%))* |  |  |
| - - Pneumonia | 1 (14.29) | 1 (6.25) |
| - - Urosepsis | 4 (57.14) | 13 (81.25) |
| - - Catheter infection | 0 (0) | 1 (6.25) |
| - - Abdominal | 1 (14.29) | 1 (6.25) |
| - - Unclear | 1 (14.29) | 0 (0) |
| **Pneumonia *(n (%))*** | 11 (13.58) | 9 (16.07) |
| - Bacterial | 1 (9.09) | 4 (44.44) |
| - Fungal | 1 (9.09) | 3 (33.33) |
| - Bacterial + fungal | 1 (9.09) | 1 (11.11) |
| - Viral | 0 (0) | 1 (11.11) |
| - No pathogen detected/only radiological diagnosis | 8 (72.73) | 0 (0) |
| Postoperative wound healing disorder *(n (%))* | 5 (6.17) | 5 (8.93) |

**Table S3. Baseline characteristics of donors and patient groups receiving either antigen-specific or non-antigen specific immunoadsorption before ABOi KTx.** Average platelet counts decreased 9.1% and 7.7% after desensitization compared to pre-IA levels, with a tendency of a lower average count in sIA. ^+^One patient underwent simultaneous appendectomy and cyst kidney nephrectomy. ^++^One patient underwent simultaneous kidney transplant nephrectomy and native nephrectomy. IA: immunoadsorption; IgG: immunoglobulin G; IgM, immunoglobulin M; KTx: kidney transplantation; PPh: plasmapheresis. Median values are provided (95% CI of median) unless indicated otherwise.

| **BASELINE CHARACTERISTICS** | | | **Antigen-specific IA** | **Non-antigen specific IA** |
| --- | --- | --- | --- | --- |
| **RECIPIENTS’ CHARACTERISTICS** | | | | |
| **Primary disease *(n (%))*** | | |  |  |
| - Glomerulonephritis | | | 37 (45.68) | 31 (54.39) |
| - Polycystic kidney disease | | | 21 (25.93) | 13 (22.81) |
| - Congenital/obstructive | | | 7 (8.64) | 2 (3.51) |
| - Hypertension | | | 1 (1.23) | 3 (5.26) |
| - Unclear | | | 6 (7.41) | 3 (5.26) |
| - Diabetic nephropathy | | | 0 (0) | 1 (1.75) |
| - Other | | | 9 (11.11) | 4 (7.02) |
| **No. of transplantations > 1 *(n (%))*** | | | 12 (14.81) | 6 (10.53) |
| - First transplantation | | | 69 (85.19) | 51 (89.47) |
| - Second transplantation | | | 11 (13.58) | 6 (10.53) |
| - Third transplantation | | | 1 (1.23) | 0 (0) |
| **Dialysis before transplantation *(n (%))*** | | | 67 (82.72) | 41 (71.93) |
| - Only haemodialysis | | | 59 (72.84) | 32 (56.14) |
| - Only peritoneal dialysis | | | 4 (4.94) | 8 (14.04) |
| - Hemo- and peritoneal dialysis | | | 4 (4.94) | 1 (1.75) |
| **Duration of dialysis before transplantation *(months)*** | | | 25 (17, 35) | 17 (12, 27) |
| - Haemodialysis | | | 23 (14, 34) | 16 (8, 24) |
| - Peritoneal dialysis | | | 25 (10, 48) | 40 (17, 54) |
| **No. of HLA mismatches A + B + DR** | | | 4 (3, 4) | 4 (3, 4) |
| - A mismatches | | | 1 (1, 1) | 1 (1, 2) |
| - B mismatches | | | 2 (1, 2) | 1 (1, 2) |
| - DR mismatches | | | 1 (1, 1) | 1 (1, 1) |
| **Blood Group (donor 🡪 recipient*) (n (%))*** | | |  |  |
| - 0 | 🡪 | B | 0 (0) | 1 (1.75) |
| - A° | 🡪 | 0 | - | 2 (3.51) |
| - A1 | 🡪 | 0 | 31 (38.27) | 20 (35.09) |
| - A2 | 🡪 | 0 | 13 (16.05) | 1 (1.75) |
| - A° | 🡪 | B | 1 (1.23) | - |
| - A1 | 🡪 | B | 5 (6.17) | 8 (14.04) |
| - A2 | 🡪 | B | 4 (4.94) | 1 (1.75) |
| - B | 🡪 | 0 | 11 (13.58) | 10 (17.54) |
| - B | 🡪 | A | 6 (7.41) | 5 (8.77) |
| - A1B | 🡪 | 0 | 1 (1.23) | 0 (0) |
| - A2B | 🡪 | 0 | 0 (0) | 1 (1.75) |
| - AB | 🡪 | A | 6 (7.41) | 7 (12.28) |
| - AB° | 🡪 | B | 1(1.23) | - |
| - A1B | 🡪 | B | 1 (1.23) | 1 (1.75) |
| - A2B | 🡪 | B | 1 (1.23) | 0 (0) |
| **SURGICAL DATA** | | | | |
| **Patients undergoing simultaneous surgical procedures *(n (%))*** | | | 27^+^ (33.33) | 16^++^ (28.07) |
| - Appendectomy | | | 3 (3.70) | 0 (0) |
| - Cyst kidney nephrectomy | | | 17 (20.99) | 10 (17.54) |
| - Kidney transplant nephrectomy | | | 7 (8.64) | 6 (10.53) |
| - Native nephrectomy (other than cyst kidneys) | | | 2 (2.47) | 1 (1.75) |
| **IMMUNOLOGICAL DATA** | | |  |  |
| **Postoperative IA** | | |  |  |
| - No. of patients needing postoperative IA *(n (%))* | | | 11 (13.58) | 5 (8.93) |
| - No. of postoperative IA when needed | | | 3 (1, 4) | 1 (1, 3) |
| **Postoperative PPh *(n (%))*** | | |  |  |
| - No. of patients needing postop. PPh | | | 0 (0) | 1 (1.75) |
| - No. of postoperative PPh | | | 0 | 2 |
| **Preoperative isoagglutinin titers** | | |  |  |
| - IgM isoagglutinin titer before Rituximab | | | 1:16 (1:16, 1:32) | 1:16 (1:8, 1:32) |
| - IgG isoagglutinin titer before Rituximab | | | 1:64 (1:32, 1:128) | 1:64 (1:32, 1:128) |
| - IgM isoagglutinin titer before first IA | | | 1:16 (1:8, 1:32) | 1:16 (1:8, 1:32) |
| - IgG isoagglutinin titer before first IA | | | 1:64 (1:32, 1:128) | 1:64 (1:16, 1:128) |
| - IgM isoagglutinin titer before KTx | | | 1:1 (0, 1:1) | 0 (0, 0) |
| - IgG isoagglutinin titer before KTx | | | 1:2 (1:1, 1:2) | 1:2 (0, 1:2) |
| **Postoperative isoagglutinin titers** | | |  |  |
| - IgM isoagglutinin titer 7 d after KTx *(median [min-max])* | | | 0 (0 – 1:16) | 0 (0 – 1:4) |
| - IgG isoagglutinin titer 7 d after KTx | | | 1:2 (1:1, 1:2) | 1:1.5 (1:1, 1:2) |
| - IgM isoagglutinin titer 14 d after KTx *(median [min-max])* | | | 0 (0 – 1:16) | 0 (0 – 1:4) |
| - IgG isoagglutinin titer 14 d after KTx | | | 1:2 (1:1, 1:2) | 1:2 (1:1, 1:4) |
| platelet count before IA | | | 209 (197, 223) | 224 (190, 241) |
| platelet count after IA | | | 196 (180, 209) | 208 (180, 224) |

**Table S4. Comparison of non-infectious complications after ABOi KTx between groups receiving either antigen-specific and non-antigen specific IA.** IA: immunoadsorption; KTx: kidney transplantation; TRAST: transplant renal artery stenosis. Median values are provided (95% CI of median) unless indicated otherwise.

| **NON-INFECTIOUS COMPLICATIONS** | **Antigen-specific IA** | **Non-antigen specific IA** |
| --- | --- | --- |
| **Complication requiring operative revision *(n (%))*** | 34 (41.98) | 21 (36.8) |
| - Days from KTx to first operative revision | 11 (3, 18) | 15.5 (8, 58) |
| - Patients needing >1 operative revision *(n (%))* | 2 (6.06) | 4 (19.05) |
| **Lymphocele *(n (%))*** | 23 (28.4) | 12 (21.05) |
| - Needing operative revision *(n (%))* | 14 (60.87) | 8 (66.67) |
| - Days from KTx to operative revision | 20.5 (15, 54) | 20 (12, 62) |
| **Postoperative bleeding/hematoma *(n (%))*** | 19 (23.46) | 12 (21.05) |
| - Haemorrhagic shock *(n (%))* | 1 (1.24) | 3 (5.26) |
| - Needing operative revision *(n (%))* | 13 (68.42) | 7 (58.33) |
| - Days from KTx to operative revision | 2 (1, 3) | 7 (1, 14) |
| **Postoperative vessel occlusion/TRAST *(n (%))*** | 4 (4.94) | 2 (3.51) |
| - Requiring operative/interventional revision *(n (%))* | 4 (100) | 1 (50) |
| - Days from KTx to operative/interventional revision | 3 (0, 73) | 76 |
| Urinoma/urinary leakage *(n (%))* | 2 (2.47) | 4 (7.14) |
| Micturition disturbance *(n (%))* | 6 (7.41) | 4 (7.14) |
| Post-transplant diabetes *(n (%))* | 3 (3.70) | 7 (12.50) |
| Postoperative Ogilvie syndrome *(n (%))* | 0 (0) | 4 (7.14) |

**Figure S2. IgM and IgG titer course pre and post transplantation.** Presented is the titer course from the administration of Rituximab 30 days prior to KTx until the 14^th^ postoperative day. The start of IA treatments 8 days before the scheduled KTx is indicated. **A:** Comparison of IgM isoagglutinin titer course between sIA and nsIA. Preoperatively, the course of isoagglutinin titers did not differ between the two groups, except for a significantly higher IgM titer in sIA directly before KTx (1:1 (0, 1:1) vs. 0 (0, 0)). Moreover, IgM titers 7 and 14 days after transplantation were significantly higher in sIA (day 7: 0 (0-1:16) vs. 0 (0-1:4) (median [min-max]); day 14: 0 (0-1:16) vs. 0 (0-1:4) (median [min-max]); also see Supplementary Material Table S3.

**B:** IgG isoagglutinin titer course was comparable between sIA and nsIA. Titers are depicted as median with 95% CI. IA: immunoadsorption; IgG: immunoglobulin G; IgM, immunoglobulin M; KTx: kidney transplantation.

**Figure S3. Pathogens causing infectious postoperative complications. A:** all detected pathogens. **B:** Pathogens causing severe postoperative infections. **Box:** Pathogens according to focus of severe infection.

**Table S5. Antibiotic susceptibility profiles for multi-drug resistant pathogens.**

| **PATHOGEN** | Amino-glycosides | Carba-penems | Cephalo-sporins | Fluor-quinolones | Glyco-peptides | Oxazol-idinones | Penicillns |
| --- | --- | --- | --- | --- | --- | --- | --- |
| E. coli ESBL | S | R | R | R |  |  | R |
| E. coli ESBL | S | R | R | R |  |  | R |
| E. coli ESBL | S | S | R | R |  |  | R |
| E. faecium VRE | R | R | R | R | R | S | R |
| E. coli ESBL | S | S | R | R |  |  | R |
| E. coli ESBL | S | S | R | R |  |  | R |
| Proteus vulgaris ESBL | S | S | R | R |  |  | R |
| E. coli ESBL | S | S | R | R |  |  | R |
| E. coli 3-MRGN | S | S | R | R |  |  | R |
| E. coli ESBL | S | S | R | R |  |  | R |
| E. faecium |  | R | R | R | S | S | R |
| E. faecium VRE |  | R | R | R | R | S | R |
| K. pneumoniae 3-MRGN | S | S | R | R |  |  | R |
| C. freudii 3-MRGN | S | S | R | R |  |  | R |
| E. coli ESBL | R | S | R | R |  |  | R |
| E. faecium VRE |  | R | R |  | R | S | R |

**Table S6. Comparison of non-infectious complications after ABOi KTx between groups receiving either antigen-specific and non-antigen specific immunoadsorption.** ^+^7 sIA patients and 3 nsIA patients suffered from more than one episode of rejection. ABMR: antibody-mediated rejection; IA: immunoadsorption; KTx: kidney transplantation; TCMR: T-cell mediated rejection. Median values are provided (95% CI of median) unless indicated otherwise.

| **OUTCOME** | **Antigen-specific IA** | **Non-antigen specific IA** |
| --- | --- | --- |
| Creatinine at discharge *(mg/dL)* | 1.5 (1.4, 1.6) | 1.5 (1.3, 1.6) |
| Creatinine at last follow-up *(mg/dL)* | 1.4 (1.3, 1.5) | 1.5 (1.3, 1.6) |
| **COMPLICATIONS** |  |  |
| Delayed graft function *(n (%))* | 5 (6.17) | 0 (0) |
| **Total no. of patients with graft rejection *(n (%))^+^*** | 24 (29.63) | 8 (14.04) |
| - ABMR | 10 (12.35) | 3 (5.26) |
| - TCMR | 11 (13.58) | 4 (7.02) |
| - Borderline | 13 (16.05) | 5 (8.77) |
| No. of biopsies | 1 (1, 2) | 1 (0, 1) |
| **Graft loss requiring re-start of dialysis *(n (%))*** | 9 (11.11) | 3 (5.26) |
| - Duration until re-start of dialysis *(days)* | 1691 (142, 3952) | 210 (13, 823) |
| - Graft loss within first 2 years after KTx *(n (%))* | 3 (33.33) | 2 (66.67) |
| - Cause of graft loss *(n (%))* |  |  |
| - - Infectious | 0 (0) | 1 (50) |
| - - Thrombotic/insufficient perfusion | 1 (33.33) | 1 (50) |
| - - Chronic rejection | 2 (66.67) | 0 (0) |
| **Death *(n (%))*** | 5 (6.17) | 5 (8.77) |
| - Death with functioning graft | 4 (80.0) | 3 (60.0) |
| - Death within first 2 years after KTx | 1 (20.0) | 5 (100.0) |
| - - with functioning graft | 0 (0) | 3 (60.0) |
| - Cause of death *(n (%))* |  |  |
| - - Infection | 3 (60) | 2 (40) |
| - - Cancer | 1 (20) | 1 (20) |
| - - Cardiovascular | 0 (0) | 2 (40) |
| - - Unknown | 1 (20) | 0 (0) |

**Table S7. Rejection episodes and their individual treatment after ABOi KTx.** Where available, biopsy samples were assessed according to Banff 2013 criteria. After ABOi KTx, unspecific C4d-positivity is commonly encountered and therefore no significant result. “x”: parameter was not interpretable due to indecisive biopsy specimen.

| **REJECTIONS** | **Banff Classification** | **Treatment** |
| --- | --- | --- |
| **HUMORAL REJECTIONS** | | |
| **ANTIGEN-SPECIFIC IA** | | |
| Patient # 1 | t1, i0, g0, ptc0, ah2, v0, cg2, ci1, ct1, cv0, mm3, c4d2, aah3 | Rituximab |
| Patient # 3 | “Tubulitis” | No information available |
| Patient # 7 | t0 i0 g2 ah0 v0 ci0 cg0 cv0 ct0 mm0 | Plasmapheresis, cortisone pulse therapy, Rituximab |
| Patient # 10 | “Tubulitis“ | Cortisone pulse therapy and quadruple therapy (tacrolimus, sirolimus, MFA, cortisone) |
| Patient # 11 | t1, i0, g1, ptc0, ah3, v0, cgx, ci1, ct1, cv2, mm1, c4d3, aah3 | Elevation of tacrolimus target trough level to 6-8 ng/ml |
| Patient # 22 | „Tubular damage and dilated peritubular capillaries with mononuclear cells“ | Cortisone pulse therapy |
| Patient # 25 | “Humoral rejection” | Plasmapheresis, ATG |
| Patient # 63 | t0, i0, g1, ptc0, ah1, v0, cg0, ci0, ct0, cv0, mm0, c4d3, aah0 | Plasmapheresis |
| Patient # 65 | t0, i0, g0, ptc0, ah0, v1, cg0, ci0, ct0, cv0, mm0, c4d3, aah0 | Plasmapheresis, ATG, tacrolimus target trough level 10-12 ng/ml for 3 months |
| Patient # 68 | t0, i0, g0, ptc2, ah1, v0, cg0, ci0, ct0, cv1, mm0, c4d3, aah0 | Plasmapheresis, ATG |
| **NON-ANTIGEN-SPECIFIC IA** | | |
| Patient # 89 | t0, i0, g1, ptc2, ah0, v1, cg0, ci0, ct0, cv0, mm0, C4d0, aah0 | Plasmapheresis, ATG |
| Patient # 115 | 05.12.16: t0, i0, g2, ptc2, ah0, v0, cg0, ci0, ct1, cv0, mm0, c4d3, aah0, ti0 | ATG |
| Patient # 122 | 12.07.17: t0, i0, g1, ptc0, ah1, v2, cg0, ci0, ct1, cv1, mm0, c4d3, aah1  21.07.17: t3, i0, g1, ptc1, ah0, v0, cg2, ci0, ct1, cv1, mm1, c4d3, aah0, ti0  21.08.17: ti0 t1, i0, g2, ptc2, ah0, v2, cg2, ci0, ct0, cv1, mm1, c4d2, aah0, ti0  13.09.17: t1, i1, g2, ptc0, ah0, v1, cg2, ci0, ct1, cv0, mm1, c4d, aah0,  24.10.17: t3, i0, g1, ptc0, ah0, v0, cg3, ci0, ct1, cv1, mm0, c4d3, aah0, ti0  07.11.17: t0, i1, g0, ptc0, ah0, v0, cg3, ci0, ct1, cv1, mm0, c4d3, aah0, ti0  27.12.17: t2, i1, g0, ptc0, ah0, v0, cg3, cix, ct1, cv1, mm1, c4d0, aah0, ti1 | Cortisone pulse therapy, Eculizumab, plasmapheresis |
| **CELLULAR REJECTIONS** | | |
| **ANTIGEN-SPECIFIC IA** | | |
| Patient # 22 | „Mild acute cellular rejection, Banff 2A“ | No information available |
| Patient # 23 | “Cellular rejection” | Plasmapheresis, ATG, cortisone pulse therapy |
| Patient # 25 | „Proliferating graft vasculopathy with subtotal luminal occlusion” | No information available |
| Patient # 26 | v1 | Cortisone pulse therapy, tacrolimus target trough level 7-9 ng/ml |
| Patient # 29 | t0, i0, g0, ptc0, ah1, v0, cg0, ci1, ct1, cv1, mm0, ah1 | No information available |
| Patient # 35 | „Granulocytic and lymphohistiocytic infiltrates with destructive tubulitis” | No information available |
| Patient # 38 | t3, i2, g0, ptc0, ah0, v0, cg0, ci0, ct1, cv0, mm0 | BK nephropathy: reduction of tacrolimus and mycophenolate, later switch to Avara |
| Patient # 60 | t1, i0, g0, ptc0, ah1, v1, cg0, ci0, ct1, cv1, mm0, c4d3, aah0 | Cortisone pulse therapy and ATG |
| Patient # 65 | t0, i0, g0, ptc0, ah0, v2, cg0, ci0, ct0, cv0, mm0, C4d3, aah0 | Plasmapheresis, ATG, tacrolimus target trough level 10-12 ng/ml for 3 months |
| Patient # 68 | t0, i0, g0, ptc2, ah1, v0, cg0, ci0, ct0, cv0, mm0, c4d, aah0. | Plasmapheresis, cortisone pulse therapy |
| Patient # 71 | t2, i1, g0, ptc0, ah0, v0, cg0, ci1, ct1, cv0, mm0, c4d3, aah0 | Cortisone pulse therapy |
| **NON-ANTIGEN-SPECIFIC IA** | | |
| Patient # 89 | t0, i1, g0, ptc1, ah0, v2, cg0, ci0, ct1, cv0, mm0, c4d0, aah0 | No information available |
| Patient # 115 | 30.12.16: t3, i3, g2, ptc3, ah0, v3, cg0, ci0, ct1, cv0, mm0, c4d2, aah0, ti3 | Dialysis |
| Patient # 123 | t3, i2, g0, ptc0, ah0, v0, cg0, ci1, ct1, cv0, mm1, c4d3, aah0, ti1 | Cortisone pulse therapy |
| Patient # 136 | t2, i1, vx, g1, ptc0, ti1, i-IFTA1, C4d1, cg0, mm0, ah0, aah0, cvx, ci0, ct1 | No information available |

**Table S8. Cause of graft failure after ABOi KTx.** In the case of recurring acute graft failures, time from KTx to the first episode is reported.

| **GRAFT FAILURE** | **Days after KTx** | **Cause** |
| --- | --- | --- |
| **ANTIGEN-SPECIFIC IA** | | |
| **ACUTE GRAFT FAILURE, RECOVERED** | | |
| Patient # 17 | 49 | Prerenal acute on chronic graft failure |
| Patient # 22 | 72 | Acute graft failure due to lymphocele formation |
| Patient # 34 | 104 | Prerenal acute graft failure |
| Patient # 41 | 1672 | Acute, infection-triggered graft failure |
| Patient # 50 | 3740 | Recurring prerenal graft failures |
| Patient # 58 | 2015 | Prerenal acute graft failure due to urosepsis |
| Patient # 65 | 1858 | Recurring acute graft failures |
| **ACUTE GRAFT FAILURE, NOT RECOVERED** | | |
| Patient # 6 | 112 | Septic multi-organ failure with acute graft loss |
| Patient # 47 | 7 | Acute graft loss due to arterial graft thrombosis with graft ischemia |
| Patient # 62 | 1691 | Acute graft loss due to urosepsis |
| **CHRONIC GRAFT FAILURE** | | |
| Patient # 1 | 4743 | Graft glomerulopathy, chronic ABMR |
| Patient # 10 | 2265 | Chronic vascular rejection, CNI toxicity |
| Patient # 11 | 3952 | Chronic ABMR |
| Patient # 51 | 597 | Graft glomerulopathy |
| Patient # 63 | 142 | Graft glomerulopathy |
| Patient # 68 | 1604 | BKV nephropathy, chronic rejection (ABMR and TCMR) |
| Patient # 71 | 1985 | Unclear, most likely chronic TCMR |
| **NON-ANTIGEN-SPECIFIC IA** | | |
| **ACUTE GRAFT FAILURE, RECOVERED** | | |
| Patient # 95 | 33 | Acute infection-triggered graft failure |
| Patient # 99 | 1042 | Prerenal acute graft failure |
| Patient # 109 | 56 | Recurring prerenal acute graft failures |
| Patient # 113 | 36 | Postrenal acute graft failure due to lymphocele |
| Patient # 116 | 358 | Acute graft failure due to diarrhoea |
| Patient # 122 | 135 | Suspected CNI-associated DD aHUS-associated acute graft failure |
| **ACUTE GRAFT FAILURE, NOT RECOVERED** | | |
| Patient # 108 | 210 | Acute graft loss due to urosepsis with coagulopathy/DIC |
| Patient # 115 | 13 | Acute graft loss due to haemorrhagic shock |
| Patient # 136 | 385 | Acute on chronic graft failure, most likely cardiorenal |
| Patient # 137 | 50 | Acute graft loss due to septic multi-organ failure |
| **CHRONIC GRAFT FAILURE** | | |
| Patient # 90 | 823 | Graft IgA-nephritis |

**Table S9. Causes of death with functioning graft for all patients after ABOi KTx.**

| **DEATH WITH FUNCTIONING GRAFT** | **Days after KTx** | **Cause** |
| --- | --- | --- |
| **ANTIGEN-SPECIFIC IA** | | |
| Patient # 14 | 4497 | Septic shock with multi-organ failure |
| Patient # 41 | 2426 | unknown |
| Patient # 58 | 2032 | Metastatic squamous cell carcinoma |
| Patient # 15 | 4986 | Septic shock with multi-organ failure |
| **NON-ANTIGEN-SPECIFIC IA** | | |
| Patient # 101 | 601 | Metastatic squamous cell lung carcinoma |
| Patient # 106 | 1 | Fatal myocardial infarction |
| Patient # 129 | 10 | Septic shock with multi-organ failure |
